# Solubility, speciation and local environment of chlorine in zirconolite glass–ceramics for the immobilisation of plutonium residues†

**DOI:** 10.1039/d0ra04938g

**Published:** 2020-09-02

**Authors:** Stephanie M. Thornber, Lucy M. Mottram, Amber R. Mason, Paul Thompson, Martin C. Stennett, Neil C. Hyatt

**Affiliations:** Immobilisation Science Laboratory, Department of Materials Science & Engineering, The University of Sheffield Sir Robert Hadfield Building, Mappin Street Sheffield S1 3JD UK n.c.hyatt@sheffield.ac.uk; XMaS, UK CRG, ESRF 71 Avenue des Martyrs 38043 Grenoble France; Department of Physics, University of Liverpool, Oliver Lodge Laboratory Liverpool L69 7ZE UK

## Abstract

The immobilisation and disposal of fissile materials from civil and defence nuclear programmes requires compatible, passively safe and proliferation resistant wasteforms. In this study, we demonstrate the application of an albite glass–zirconolite ceramic material for immobilisation of chloride contaminated plutonium oxide residues in the United Kingdom. The chlorine solubility limit in the albite glass phase was determined to be 1.0 ± 0.1 wt%, above the maximum envisaged chorine inventory of 0.5 wt%, attainable at a 20 wt% PuO_2_ incorporation rate within the ceramic. Cl K-edge of X-ray Absorption Near Edge Spectroscopy (XANES) was exploited to confirm partitioning of Cl to the glass phase, speciated as the chloride anion, with exsolution of crystalline NaCl above the chlorine solubility limit. Combinatorial fitting of Cl XANES data, utilising a library of chemically plausible reference spectra, demonstrated the association of Cl with Na and Ca modifier cations, with environments characteristic of the aluminosilicate chloride minerals eudialyte, sodalite, chlorellestadite and afghanite. Adventitious incorporation of Ca, Zr and Ti within the albite glass phase apparently assists chlorine solubility, by templating a local chemical environment characteristic of the mineral reference compounds. The partitioning of Ce, as a Pu analogue, within the glass–ceramic was not adversely impacted by incorporation of Cl. The significance of this research is in demonstrating the compatibility of the glass–ceramic wasteform toward Cl solubility at the expected incorporation rate, below the determined solubility limit. Thus, an upstream heat treatment facility to remove chloride contamination, as specified in the current conceptual flowsheet, would not be required from the perspective of wasteform compatibility, thus providing scope to de-risk the technology roadmap and reduce the projected capital and operational plant costs.

## Abstract 2

Understanding chlorine speciation and incorporation in glass ceramic wasteforms underpins future treatment of contaminated plutonium residues.

## Graphical abstract

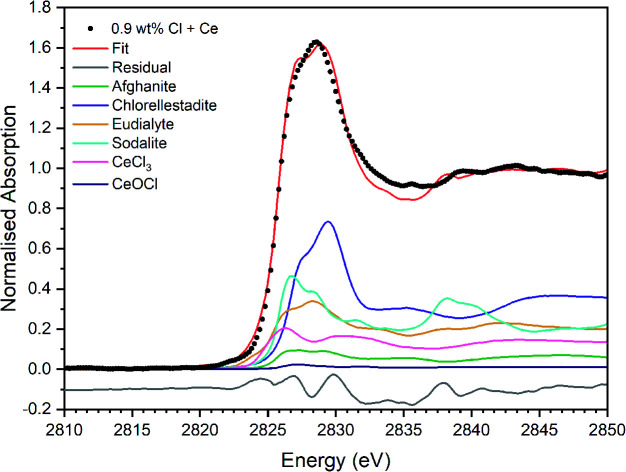

## 1. Introduction

The UK holds the largest stockpile of civil separated plutonium projected to exceed 140 tons at the end of reprocessing, stored as PuO_2_.^1^ Current Government policy is for UK plutonium to be reused as mixed oxide (MOX) fuel in civil nuclear reactors, with any material unsuitable for reuse to be immobilised as a waste for geological disposal.^1,2^ At present, this policy is challenged by lack of commercial interest in MOX off take by reactor operators. In the event that reuse of plutonium as MOX fuel cannot be delivered, immobilisation of the stockpile could be required since regulators require conversion of PuO_2_ powder into an alternative passive form more suitable for long term storage.^1^

Separated plutonium unsuitable and uneconomic for reuse includes PuO_2_ residues arising from early plutonium based research and development, secondary reprocessing wastes, and unused MOX materials.^3–5^ Powder PuO_2_ residues are contained within PVC (polyvinyl chloride) packaging, within stainless steel or aluminium containers. Some PVC packaging has degraded during storage, as a consequence of radiogenic heating and radiolysis, which has resulted in chloride contamination of the PuO_2_.^6,7^ The upper bound of Cl contamination for PuO_2_ residues is estimated at *ca.* 2.0 wt%, although with considerable uncertainty and variation between packages.^6,7^ Typical expected Cl levels before heat treatment are in the range of 0.5–1.0 wt%, thus the upper bound of 2.0 wt% is very conservative in our assumptions. A zirconolite glass–ceramic wasteform, manufactured by hot isostatic pressing (HIPing) has been developed as a flexible wasteform for the immobilisation of plutonium residues and stockpile material.^3–12^ In this wasteform, Pu is targeted for solid solution in the zirconolite ceramic phase, prototypically CaZrTi_2_O_7_, and the accessory albite glass phase, prototypically NaAlSi_3_O_8_, acts to incorporate feed impurities. Zirconolite was selected as the plutonium host phase due to its known chemical durability, radiation tolerance and demonstrable retention of actinides over geological timescales in mineral counterparts, see *e.g.*ref. 13 for a comprehensive review. We have previously demonstrated a formulation and processing route to yield zirconolite glass–ceramics, targeting 70 wt% zirconolite and 30 wt% NaAlSi_3_O_8_, with efficient plutonium and cerium surrogate partitioning between the zirconolite and glass phases (100 : 1 and 20 : 1 respectively), and only trace accessory crystalline phases present (*e.g.* ZrSiO_4_, CaTiSiO_5_, TiO_2_).^14,15^

Cl contamination of PuO_2_ residues could pose a challenge to the formulated zirconolite glass–ceramic wasteform. The zirconolite structure is not known to incorporate the chloride anion, whereas Cl solubility in alkali/alkaline earth aluminosilicate glasses is typically less than a few weight percent; above this threshold, phase separation of an alkali or alkaline earth chloride occurs.^16–20^ In respect of the composition of the current wasteform, phase separation of NaCl, and potentially PuCl_3_, is conceivable since there is no evidence of solid solution formation;^21^ this would clearly be undesirable given the aqueous solubility of PuCl_3_. In addition, Cl incorporation has been shown to result in a small, but measurable, increase in aluminosilicate melt viscosity.^22–24^ Addition of 1.1 mol% Cl (0.6 wt%) in Na_2_O–CaO–Al_2_O_3_–SiO_2_ melts was reported to increase viscosity by a factor of 10 in peralkaline melts, whereas addition of 0.6 mol% Cl (0.3 wt%) in peraluminous melts was reported to reduce viscosity by a factor of 3.^25^ In the context of this study, the viscosity of the glass component will influence the kinetics of diffusion and hence zirconolite formation.

The current conceptual process for HIP immobilisation of plutonium residues incorporates provision for a heat treatment facility to remove Cl contaminants prior to immobilisation, due to the uncertainty of Cl behaviour within the wasteform.^26^ The flowsheet and delivery plan for immobilisation technology could be significantly de-risked if the requirement for a heat treatment plant were removed and satisfactory incorporation of the Cl inventory within the wasteform assured. Consequently, a robust safety case for the wasteform should include a determination of the Cl solubility limit in the glass phase and knowledge of the impact on the desired phase assemblage. This requires a mechanistic understanding of Cl incorporation at the atomic scale. A previous preliminary study of Cl incorporation in a non-optimised glass–ceramic formulation demonstrated satisfactory incorporation in excess of the expected level in the feed material, though the mechanism and limit of Cl solubility were not determined.^7^

More broadly, the incorporation mechanism of Cl in aluminosilicate melts is of considerable significance for the global chlorine cycle, since magmas transfer Cl from the mantle to the crust and atmosphere, and dissolved hydrosaline liquids have an important role in subsequent ore formation.^27–29^ Cl solubility in aluminosilicate melts is known to depend on composition, temperature and pressure.^19,20,27,28^^35^Cl Magic Angle Spinning-Nuclear Magnetic Resonance (MAS-NMR) studies have yielded considerable insight into Cl incorporation mechanisms in aluminosilicate glasses. These studies have demonstrated co-ordination of Cl^−^ by alkali/alkaline earth modifier cations, with no discernible evidence for significant Al–Cl or Si–Cl bonds.^30–32^ However, ^35^Cl MAS-NMR is challenging since ^35^Cl is a spin 3/2 quadrupole nuclide, with a low resonance frequency and large quadrupole moment, resulting in relatively broad signals. Cl K-edge X-ray Absorption Spectroscopy (XAS) has not been extensively applied to understand the speciation of Cl in aluminosilicate glasses, no doubt due to the low energy of the Cl K-edge (2822.4 eV) for which there are relatively few suitable synchrotron beamlines in the tender X-ray regime. These studies are typically constrained by the need to utilise fluorescence detection due to prohibitive sample attenuation in transmission mode, leading to distortion of the signal by self-absorption effects for concentrated compounds, although this can be corrected post-measurement. Nevertheless, Cl K-edge X-ray Absorption Near Edge Spectroscopy (XANES) studies of CaO–MgO–Al_2_O_3_–SiO_2_ glasses suggested a mechanism of incorporation involving Cl^−^ co-ordinated to Mg and Ca network modifiers.^33^ Likewise, a combined XANES and EXAFS (Extended X-ray Absorption Fine Structure) study of borosilicate glasses intended for radioactive waste immobilisation suggested Cl^−^ co-ordinated to Ca.^34^

Here, we investigate the Cl solubility limit in glass–ceramics formulated to yield 30 wt% NaAlSi_3_O_8_ glass and 70 wt% CaZrTi_2_O_7_ and its effect on the phase assemblage and plutonium surrogate partitioning behaviour. Cl K-edge XAS was applied to determine the chloride speciation and incorporation mechanisms within the glass phase. Our results demonstrate a Cl solubility limit in the aluminosilicate glass phase of 1.0 ± 0.1 wt%, which would be sufficient to accommodate the conservative upper bound Cl inventory, without prior heat treatment, at the baseline waste incorporation rate of 20 wt% PuO_2_ within our glass–ceramic formulation. Assuming 2 wt% Cl contamination, a 20 wt% PuO_2_ loading within the 70 wt% ceramic fraction of the glass–ceramic would yield an upper bound Cl concentration of 1.0 wt% within the glass component of the wasteform. In reality, typical Cl concentrations are expected to be *ca.* 0.5–1.0 wt% prior to treatment, which would yield an expected upper limit Cl concentration of 0.5 wt% within the glass, thus, giving a conservative margin for accommodating the Cl inventory. Consequently, from the perspective of wasteform formulation, a heat treatment plant would not be required to reduce the Cl contamination prior to immobilisation, as in the current conceptual process flow sheet.^26^

## 2. Experimental

Prototype glass–ceramics for plutonium immobilisation were formulated to a previously optimised baseline composition,^10,12,14,15^ targeting 30 wt% glass of composition NaAlSi_3_O_8_ and 70 wt% ceramic phase CaZrTi_2_O_7_. Cl was added to the baseline formulation by replacement of Na_2_O with 2NaCl, to yield nominal Cl concentrations of 0.3, 0.6, 0.9, 1.7 and 2.5 wt%. Two additional samples were fabricated with CeO_2_ as a PuO_2_ surrogate, targeting incorporation as Ce^4+^ on the Zr^4+^ site of the zirconolite (CaZr_0.8_Ce_0.2_Ti_2_O_7_), to understand the potential association between Ce and Cl in the phase assemblage. Powder batches to yield 50 g of the compositions summarised in Table 1, were constituted from stoichiometric amounts of SiO_2_, Na_2_SiO_3_, Al_2_O_3_, CaTiO_3_, TiO_2_, ZrO_2_, CeO_2_ and NaCl. Powders were milled at 500 rpm for 30 min in a planetary mill with heptane as the milling medium (in which NaCl is insoluble). Milled powders were calcined overnight at 600 °C before packing into the HIP canisters. The canisters were evacuated at room temperature and baked-out at 300 °C before sealing and were HIPed at 1250 °C for 4 h under 103 MPa of argon gas pressure.

**Table 1:** Sample matrix. Sample compositions A–E had the same baseline formulation with increasing Cl content by replacement of Na_2_O by 2NaCl. Compositions F and G targeted Ce^4+^ incorporation on the Zr^4+^ site within the zirconolite phase

Composition	Glass–ceramic formulation	Nominal Cl wt% (added as NaCl)
A	30 wt% NaAlSi_3_O_8_	0.3
	70 wt% CaZrTi_2_O_7_	
B	30 wt% NaAlSi_3_O_8_	0.6
	70 wt% CaZrTi_2_O_7_	
C	30 wt% NaAlSi_3_O_8_	0.9
	70 wt% CaZrTi_2_O_7_	
D	30 wt% NaAlSi_3_O_8_	1.7
	70 wt% CaZrTi_2_O_7_	
E	30 wt% NaAlSi_3_O_8_	2.5
	70 wt% CaZrTi_2_O_7_	
F	30 wt% NaAlSi_3_O_8_	0.9
	70 wt% CaZr_0.8_Ce_0.2_Ti_2_O_7_	
G	30 wt% NaAlSi_3_O_8_	1.7
	70 wt% CaZr_0.8_Ce_0.2_Ti_2_O_7_	

Monolithic glass–ceramic specimens were ground and polished to study the microstructure and elemental distribution using a Hitatchi TM3030 analytical scanning electron microscope (SEM) and a Bruker Quantax Energy Dispersive X-ray Spectrometer (EDX). Compositional analysis was performed by powder X-ray diffraction (PXRD) using a Bruker D2 PHASER diffractometer with Cu Kα radiation (1.5418 Å) and a Lynxeye position sensitive detector.

XAS data were acquired on the XMaS bending magnet beamline (BM28) at the European Synchrotron Radiation Facility, Grenoble, France. The XMaS beamline was configured with a fixed exit, double crystal, Si (111) monochromator; a rhodium coated toroidal mirror of silicon crystal focused the beam to a spot size of 1 mm. Harmonic rejection was provided by rhodium coated pyrex mirrors.^35^ Cl K-edge XAS data were acquired at room temperature in fluorescence mode, using a Vortex Si drift detector, with the samples orientated at an incidence angle of 45° between the beam and detector, under a helium atmosphere.

The absolute energy scale was calibrated to the L_3_ absorption edge of a Rh reference foil set at *E*_0_ = 3004.0 eV.^36^ Since the XMaS beamline utilised an optically encoded monochromator, the energy drift between scans was expected to be negligible and this was verified by periodic acquisition of data from the Rh reference foil. A comprehensive account of the specifications for tender EXAFS measurements has been previously published.^35^

Samples for XAS analysis were prepared as 6 mm pellets of a homogenous dispersion of analyte powder in *ca.* 20 mg polyethylene glycol as a binder. Data reduction and analysis were performed using the programmes Athena, Artemis and Hephaestus.^37^ Data acquisition and analysis were restricted to the near-edge region due to the presence of trace Ar within the He gas environment and consequent absorption edge at *E*_0_ = 3207.0 eV,^36^ which prevented reliable background subtraction for analysis of the extended X-ray absorption fine structure. A library of XANES data was compiled from a suite of reference minerals and synthetic compounds, verified by XRD and qualitative EDX analysis. The mineral reference compounds (with prototypical chemical compositions, identifier, and provenance^38^) were: afghanite ((Na,Ca,K)_8_(Si,Al)_12_O_24_(SO_4_,Cl,CO_3_)_3_, NCH2017.01 – Badakhshan Province, Afghanistan); chlorellestadite (Ca_5_(SiO_4_,SO_4_,PO_4_)_3_(Cl,F), BM.2011,22 – Caspar Quarry, Eifel, Rheinland Palatinate, Germany); davyne ((Na,Ca,K)_8_Al_6_Si_6_O_24_(Cl,SO_4_,CO_3_)_2–3_, BM.94667 – Mount Vesuvius, Italy); eudialyte (Na_4_(Ca,Ce)_2_(Fe^2+^;Mn^2+^)ZrSi_8_O_22_(OH,Cl)_2_, GDUS SN4, Christiania Mine, Oslo, Norway); marialite (Na_4_AlSi_3_O_8_Cl, BM.1971,216 – Gooderham, Haliburton Co., Ontario, Canada); scapolite ((Na,Ca)_4_(Al,Si)_3_(Si_6_O_24_)(Cl,CO_3_)·H_2_O, GDUS J.6.189 – Bolton Massachusetts, USA); sodalite (Na_8_Al_6_Si_6_O_24_Cl_2_, BM.1985,79 – South Africa). The synthetic reference compounds were: NaCl, CaCl_2_, CaCl_2_·2H_2_O, Ca_3_SiO_4_Cl and Ca_12_Al_14_O_32_Cl_2_; plus CeCl_3_, CeCl_3_·7H_2_O and CeOCl.

## 3. Results

### 3.1. Chlorine incorporation in baseline compositions

#### Phase assemblage and microstructure

**Fig. 1:**
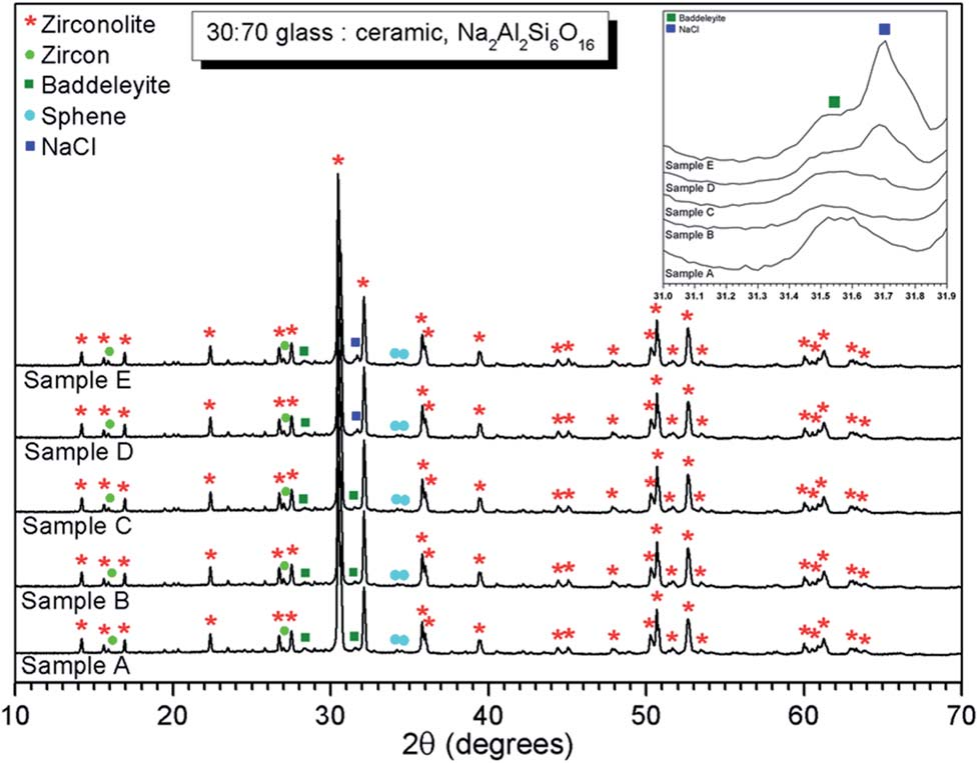
PXRD data of glass–ceramic sample compositions A–E, showing zirconolite (CaZrTi_2_O_7_) as the major crystalline phase with trace zircon (ZrSiO_4_), sphene (CaTiSiO_5_) and baddeleyite (ZrO_2_). Inset shows ingrowth of (200) reflection of NaCl, which is clearly apparent at 2*θ* = 31.7° in the data of compositions D and E.

Hot isostatic pressing of the baseline compositions in Table 1, produced high quality glass–ceramics with densities above 98% of theoretical density (Table 2). Powder XRD (PXRD) analysis confirmed zirconolite (CaZrTi_2_O_7_) was the major crystalline phase in all samples, Fig. 1, with zircon (ZrSiO_4_), sphene (CaTiSiO_5_) and baddeleyite (ZrO_2_) present in trace quantities (PDF cards: 01-074-0669, 00-006-0266, 01-076-6576 and 01-080-0966, respectively). The PXRD data of compositions D and E, with nominal 1.7 wt% and 2.5 wt% Cl, exhibited the most intense reflection of NaCl at 2*θ* = 31.7° (indexed as (2 0 0), PDF card: 00-005-0628), as shown in the inset of Fig. 1. This reflection was not present in the PXRD data of compositions A–C, with lower Cl content, implying a Cl solubility of at least 0.9 wt% in the aluminosilicate glass phase.

**Table 2:** Powder mass and canister volumes before and after processing. Pycnometry (true density) and Archimedes (bulk density) measurements were used to determine the material densification for the final HIPed materials

Composition	Powder mass (g) (±0.05)	Canister volume		Canister densification% (±0.8)	Density		Density% theoretical (±0.5)
		Before (cm^3^) (±0.4)	After (cm^3^) (±0.4)		True (g cm^−3^) (±0.01)	Bulk (g cm^−3^) (±0.04)	
A	44.41	41.9	25.8	38.3	3.57	3.54	99.3
B	34.82	34.6	21.6	37.5	3.56	3.56	99.7
C	46.63	43.2	25.8	40.3	3.57	3.51	98.6
D	45.00	42.7	25.7	39.8	3.55	3.51	99.1
E	44.43	42.8	25.7	39.9	3.51	3.51	99.8
F	49.35	45.5	27.9	38.7	3.66	3.58	97.7
G	48.09	43.9	27.4	37.7	3.58	3.56	99.4

**Fig. 2:**
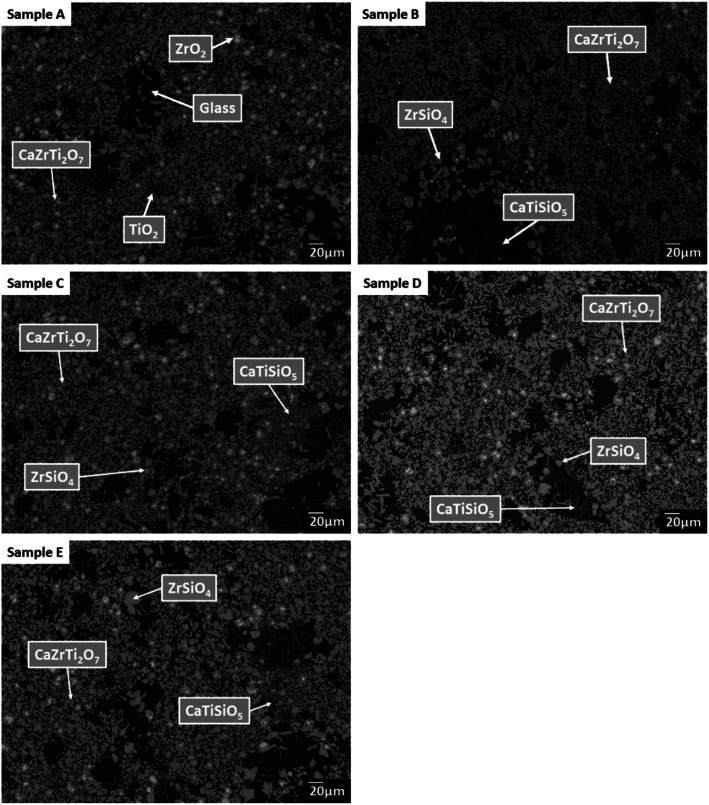
Backscattered electron micrographs of sample compositions A–E, highlighting presence of glass and zirconolite (CaZrTi_2_O_7_) phases, plus trace baddeleyite (ZrO_2_), zircon (ZrSiO_4_), sphene (CaTiSiO_5_) and rutile (TiO_2_).

**Fig. 3:**
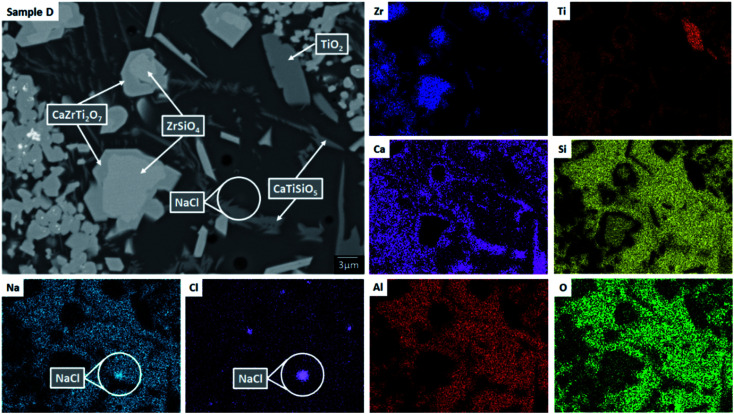
Backscattered electron micrograph and EDX maps for sample composition D (1.7 wt% Cl), highlighting hotspots of NaCl, demonstrating the Cl solubility limit in the glass phase has been exceeded. Note ZrSiO_4_ crystals surrounded by CaZrTi_2_O_7_ implying replacement of the former by the latter, *via* outward diffusion of Si/inward diffusion of Ca and Ti to/from the glass phase.

SEM-EDX analysis verified the phase assemblage determined by PXRD. The backscattered electron (BSE) micrographs in Fig. 2 show the microstructure to be comprised of homogeneously distributed crystallites of CaZrTi_2_O_7_, with trace CaTiSiO_5_, ZrSiO_4_, ZrO_2_ and TiO_2_. As shown in Fig. 3, bright ZrO_2_ cores were observed in some *ca.* 1 μm sized CaZrTi_2_O_7_ crystallites, whereas some larger, *ca.* 10 μm, crystallites showed replacement of ZrSiO_4_ by CaZrTi_2_O_7_. Thus, two distinctive dissolution–precipitation reactions are evidently involved in forming zirconolite in this system, leading to two different crystallite sizes governed by that of the ZrO_2_ and ZrSiO_4_ templating phases; an inward diffusion of Ca and Ti occurs from the melt, in conjunction with an outward diffusion of Si from ZrSiO_4_.

EDX point spectra were acquired from the zirconolite and glass phases, to investigate Cl partitioning. These analyses, shown in Fig. S1,† demonstrated Cl to be concentrated in the glass phase, with no evidence for Cl incorporation in the zirconolite phase. For compositions D and E, with nominal 1.7 wt% and 2.5 wt% Cl respectively, EDX maps showed isolated, micron sized regions of Na and Cl co-located in high concentration, as demonstrated in Fig. 3. Such an association of Na and Cl was not observed in compositions A–C, with lower Cl content. These data were consistent with the PXRD analysis, which revealed the presence of crystalline NaCl above an apparent Cl solubility limit of at least 0.9 wt% in the glass phase (Fig. 1).

**Fig. 4:**
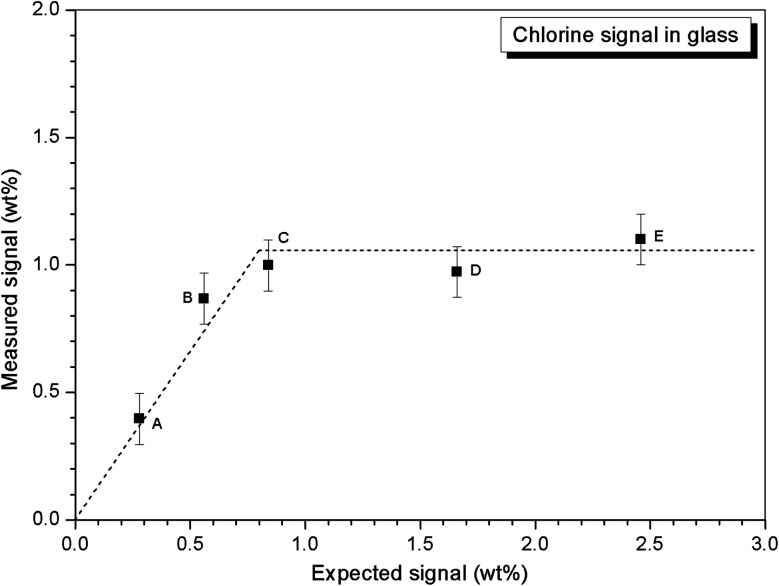
Dependence of measured Cl content in glass phase on targeted Cl content.

Quantitative EDX analysis of the glass phase, for each composition, was performed to determine the Cl solubility limit. The average compositions of the glass phases are reported in Table 3, determined from at least ten individual point analyses. Fig. 4 shows the measured Cl content of the glass phase, as a function of the expected Cl content, in wt%. A linear correlation between measured and expected content in the glass phase was apparent up to 0.9 wt% Cl, above which the measured Cl content remained independent of the amount of Cl in the batch composition. The Cl solubility limit in the glass phase, estimated from the intercept of the plateau, was determined to be 1.0 ± 0.1 wt% Cl, consistent with the phase separation of crystalline NaCl observed by PXRD and SEM-EDX of compositions D and E, with nominal 1.7 wt% and 2.5 wt% Cl, respectively. Below the Cl solubility limit, the measured Cl content in the glass phase was always slightly greater than the expected Cl content; this discrepancy would imply a small systematic inaccuracy in our EDX analysis.

**Table 3:** SEM-EDX analysed compositions of the glass phase of glass–ceramic sample compositions A–G

Oxide	Composition A		Composition B		Composition C		Composition D		Composition E		Composition F		Composition G	
	Wt%	±	Wt%	±	Wt%	±	Wt%	±	Wt%	±	Wt%	±	Wt%	±
Na_2_O	10.4	0.7	10.4	0.7	10.7	0.7	12.1	0.7	11.4	0.7	12.3	0.7	12.4	0.7
Al_2_O_3_	13.4	0.6	14.5	0.8	14.5	0.8	15.6	0.8	16.0	0.8	17.3	0.6	14.9	0.8
SiO_2_	52.0	2.1	50.0	1.9	48.8	2.1	53.5	2.1	53.4	2.1	58.1	2.1	56.6	1.9
ZrO_2_	6.1	0.3	6.0	0.3	5.7	0.3	3.1	0.1	3.1	0.1	0.6	0.3	1.2	0.3
Cl	0.4	0.1	0.9	0.1	1.0	0.1	1.0	0.1	1.1	0.1	1.0	0.1	1.1	0.1
CaO	6.1	0.3	6.9	0.3	6.9	0.3	6.5	0.3	7.2	0.3	4.7	0.3	5.1	0.3
TiO_2_	13.0	0.3	12.9	0.3	13.3	0.3	9.0	0.3	9.2	0.3	4.7	0.3	6.6	0.3
CeO_2_	—	—	—	—	—	—	—	—	—	—	0.3	0.1	0.1	0.1

#### Cl K-edge X-ray absorption near edge spectroscopy

**Fig. 5:**
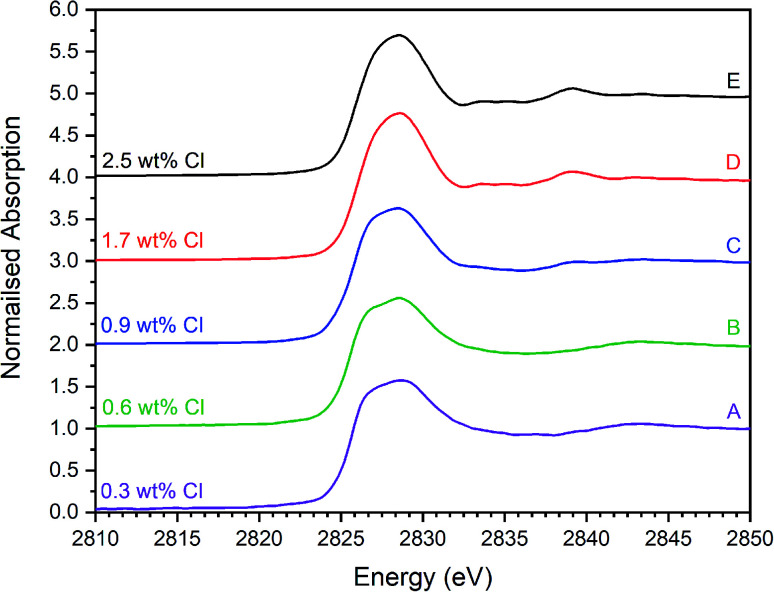
Merged, background subtracted and normalised Cl K-edge XANES data for glass–ceramic sample compositions A–E.

**Fig. 6:**
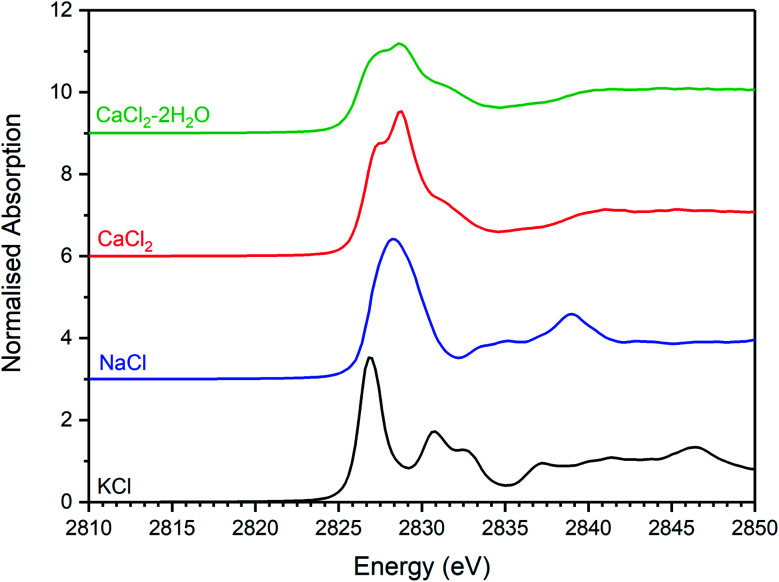
Merged, background subtracted and normalised Cl K-edge XANES data for binary chloride reference compounds.

**Fig. 7:**
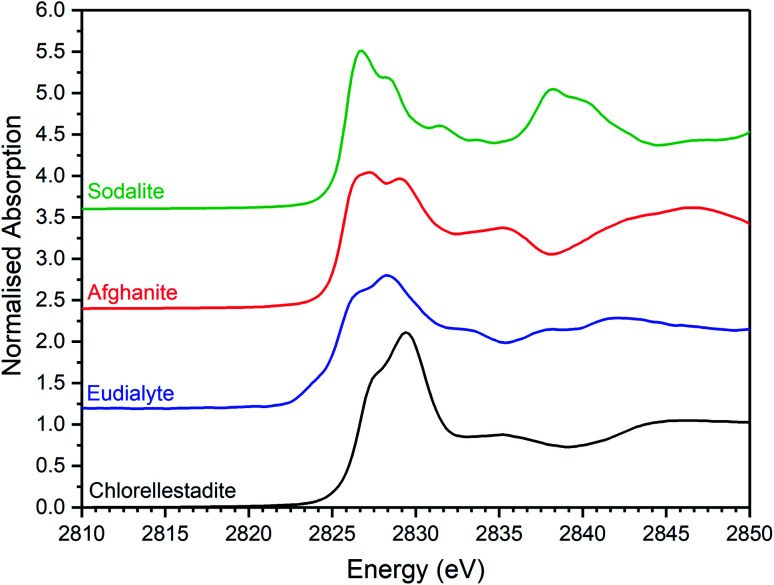
Merged, background subtracted and normalised Cl K-edge XANES data for selected aluminosilicate chloride reference compounds.

Fig. 5–7 show the merged, background subtracted, and normalised Cl K-edge XANES data for the glass–ceramic samples and selected reference compounds; a three point smoothing algorithm was applied to each data set. All data from reference compounds were corrected for self-absorption using the FLUO algorithm.^39^ For reference compounds with relatively dilute Cl concentration (*e.g.* chlorellestadite, eudialyte, sodalite, and afghanite) the impact of this correction on the relative intensity of the white line features was marginal.

The XANES features of the glass–ceramics were typically damped compared to those of the crystalline reference compounds, as shown in Fig. 5–7, from which we infer that Cl is located in one or more disordered environments within the amorphous glass phase, consistent with interpretation of SEM-EDX data. Random phase coherence of scattering paths is known to attenuate XANES features in disordered materials, relative to crystalline counterparts.^40^

The Cl K-edge XANES data of the reference compounds and glass–ceramics, shown in Fig. 5–7, all exhibited *E*_0_ in the range 2825.6–2826.8 eV, with precision ±0.2 eV (determined as the maximum of the first derivative). Since Cl is known to be speciated as the chloride anion in the reference compounds, the same characteristic *E*_0_ implied speciation as Cl^−^ in the glass–ceramic materials. The XANES data of the glass–ceramics showed a small, but systemic increase, in *E*_0_ with increased nominal Cl content, from 2825.6 eV for 0.3 wt% Cl to 2826.0 eV for 2.5 wt% Cl. Furthermore, with increased nominal Cl concentration in the glass–ceramics, a subtle change in the white line profile was apparent in the XANES data. At low Cl concentrations (≤0.9 wt%), the white line was clearly composed of two distinct features, associated with maxima at *ca.* 2827.9 and 2828.6 eV. Whereas, at high Cl concentrations (≥1.7 wt%), this distinction was no longer apparent and the features merged to give a single maximum at 2828.6 eV. These observations suggested a subtle change in Cl environment as a function of increasing Cl concentration. An additional, relatively sharp, feature was apparent at 2839 eV in the XANES data of the glass–ceramics with nominal 1.7 wt% and 2.5 wt% Cl, together with two additional subtle features in the range 2833–2835 eV. These features were also apparent at the same energy intervals in the XANES of the NaCl reference, from which it was inferred that a distinctive NaCl like environment was present in these compositions. This is consistent with the analysis of PXRD and SEM-EDX data, which demonstrated phase separation of crystalline NaCl above the solubility limit of 1.0 ± 0.1 wt% Cl in the glass phase.

Comparison of the Cl K-edge XANES data of the glass–ceramics, with those of the binary chlorides, Fig. 5 and 6, did not reveal correspondence of similar characteristic features. In addition, the *E*_0_ of the binary chlorides was typically 0.5–1.0 eV higher than that determined for the glass–ceramics. In contrast, the *E*_0_ determined for the aluminosilicate reference compounds, Fig. 7, was determined to be the in the narrow range 2825.5–2825.8 eV, comparable with that determined for the glass–ceramic materials.

Comparison of the XANES features of aluminosilicate reference compounds and glass–ceramic materials, demonstrated that no individual aluminosilicate compound was a unique fingerprint for the Cl environments in the glass–ceramics. However, the XANES data of the glass–ceramic materials evidently tracked through the XANES features of eudialyte, sodalite, and afghanite, see Fig. S2.† This suggested that the Cl environments in these aluminosilicate reference compounds could be plausible models for those in the glass–ceramic materials. Furthermore, the white lines of the XANES of the aluminosilicate reference compounds were composed of two distinct features, similar to those observed in the glass–ceramic compounds with low nominal Cl concentration, as described above. Overall, from this comparative interpretation of the XANES data of glass–ceramics and reference compounds, it was concluded that Cl was incorporated as Cl^−^ anions in the aluminosilicate glass phase, with a distribution of Cl environments which could be approximated by a combination of those characteristic of eudialyte, sodalite, and afghanite.

Combinatorial linear combination analysis was applied to the Cl K-edge XANES data of the glass–ceramic materials, with the aim of identifying and quantifying representative model Cl environments. An energy window of 2805–2850 eV was utilised for fitting the library of XANES data from 12 chemically plausible reference compounds, as summarised in Section 2.

Evaluation of all possible 2^12^ combinations of reference data was achieved using the combinatorial fitting tool in the Athena software.^37^ The fitted weighting factors of the reference spectra (*w*_*i*_) were constrained to the range 0 ≤ *w*_*i*_ ≤ 1. Since it could not be assumed that the reference library would fully account for all Cl environments in the glass–ceramic materials, the sum of the weighting factors was not constrained to unity. The goodness of fit was evaluated using the *R*-factor defined in Athena as (eqn (1)):1
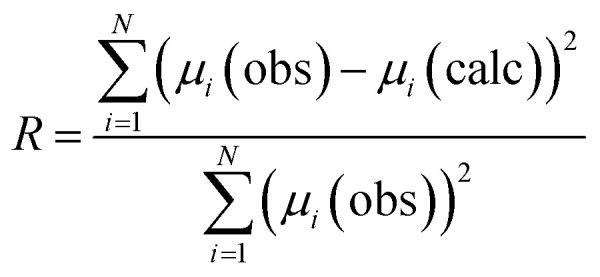
where *N* is the number of data points in the XANES spectrum, *μ*_*i*_ is normalised absorption, and obs or calc refer to the observed and calculated *μ*_*i*_ values, respectively, at each point, *i*.

The Hamilton *R*-factor ratio test was applied to compare the fit with lowest *R*-factor to each fit with progressively higher *R*-factors, applying a significance level of 0.05.^41^ This analysis afforded a subset of between 4 and 20 fits which were not significantly different in terms of goodness of fit at the 95% confidence level. The subset of fits was combined to produce a mean weighted fit of component reference data with associated uncertainties. The resulting mean weighted fits were characterised by four common contributions: eudialyte, sodalite, chlorellestadite, and afghanite, plus NaCl for the glass–ceramics with nominal 1.7 wt% and 2.5 wt% Cl. All other contributions, with a weighting factor much smaller than the associated uncertainty, were not considered significant. Fig. 10 shows the derived mean weighted contributions of XANES data from the reference compounds used to fit the glass–ceramics data, with all associated uncertainties.

**Fig. 8:**
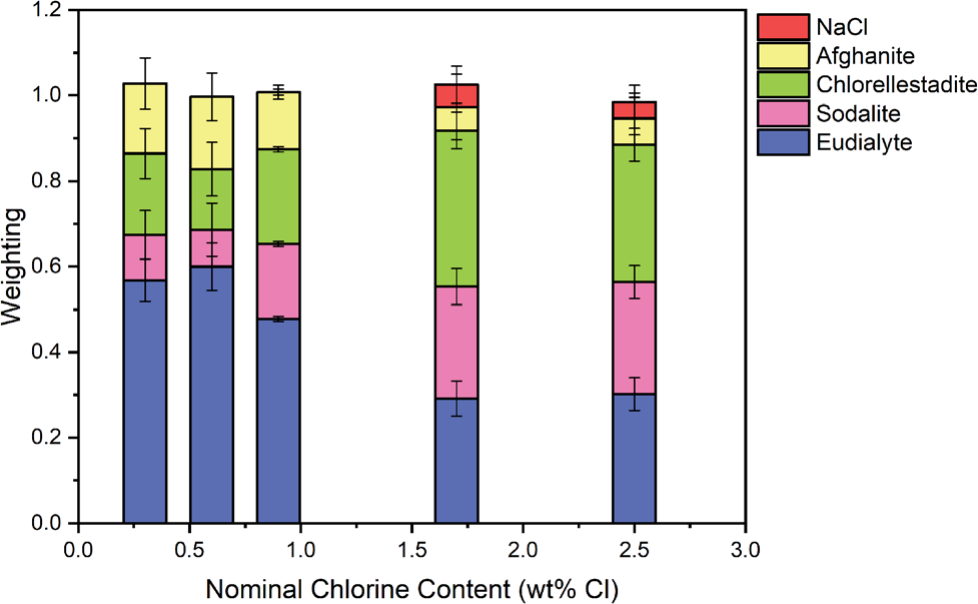
The mean weighted contributions of XANES data from reference compounds NaCl, afghanite, chlorellestadite, sodalite and eudialyte, required to fit the data of glass–ceramic sample compositions A–E; see text for discussion.

Fig. 8 shows a systematic evolution in Cl environments within the glass–ceramics with increasing Cl concentration. At low nominal Cl concentration (≤0.9 wt% Cl), the major environment is described by eudialyte, with minor contributions from sodalite, afghanite and chlorellestadite. At higher concentrations, the Cl environments are described by approximately equal proportions of eudialyte, chlorellestadite, and sodalite, with a minor contribution from afghanite and NaCl. The contribution of NaCl was present only in glass–ceramic compositions, where the nominal Cl content exceeded the solubility limit of 1.0 ± 0.1 wt% Cl in the glass phase, leading to phase separation of crystalline NaCl, as detected by PXRD and SEM-EDX. The contribution of NaCl in fitting the XANES data of these compositions was also consistent with the appearance of relatively sharp and characteristic XANES features attributed to crystalline NaCl, and the small observed increase in *E*_0_, due to the significantly higher edge shift of NaCl (2826.8 eV) relative to the glass–ceramics with lower Cl concentration (∼2825.6 eV). Thus, the NaCl environment identified from combinatorial linear combination analysis is considered to be associated with crystalline phase separated NaCl, to first order.

**Fig. 9:**
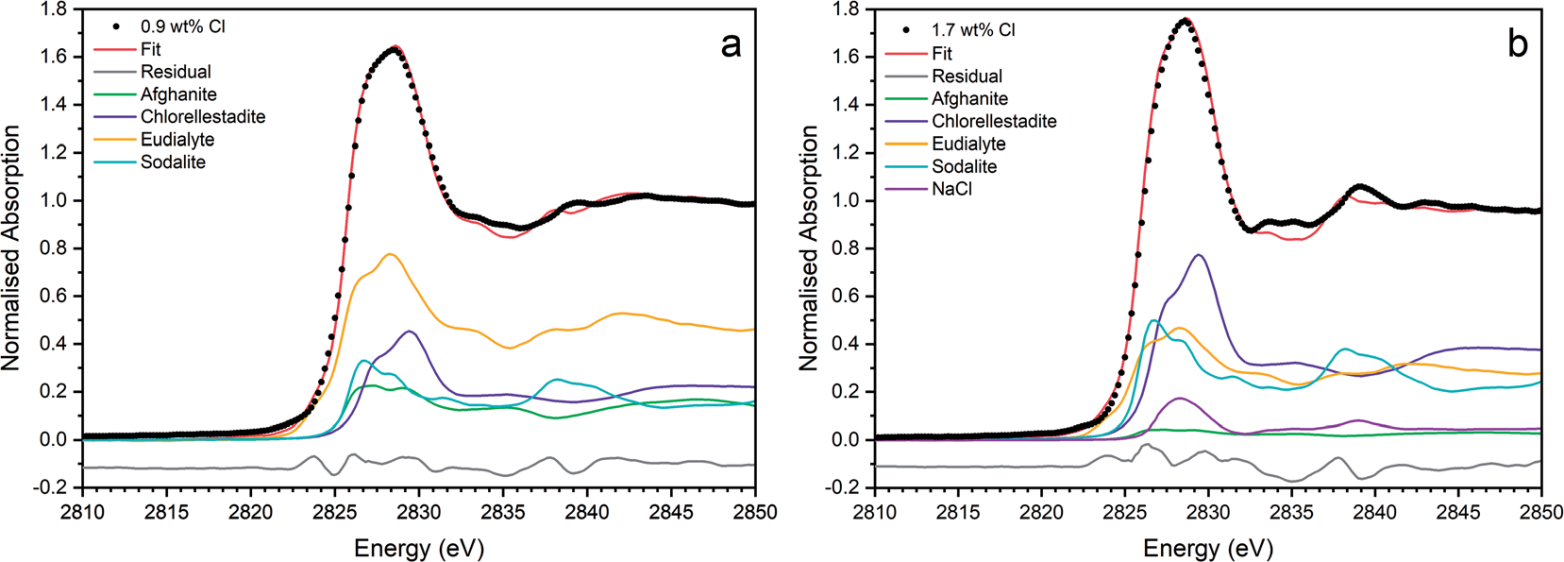
Linear combination fits with the lowest *R* factor for combinatorial fitting of reference spectra to Cl incorporated baseline glass–ceramic sample compositions with (a) 0.9 wt% Cl (composition C) and (b) 1.7 wt% Cl (composition D).

Fig. 9 shows the linear combination fits with the lowest *R* factor for the glass–ceramics with nominal 0.9 wt% and 1.7 wt% Cl. Inspection of the fit and difference profile showed that, although the sum of the four weighted reference data sets (plus NaCl) provided a reasonable fit to the observed data, one or more additional components were evidently required for a complete description. Consequently, our interpretation of the model Cl environments present in the glass–ceramics is incomplete, however, the adequacy of the fit enables consideration of the relative proportion of model Cl environments, though absolute values should be treated with due caution.

### 3.2. Chlorine incorporation in CeO_2_ doped baseline compositions

#### Phase assemblage and microstructure

**Fig. 10:**
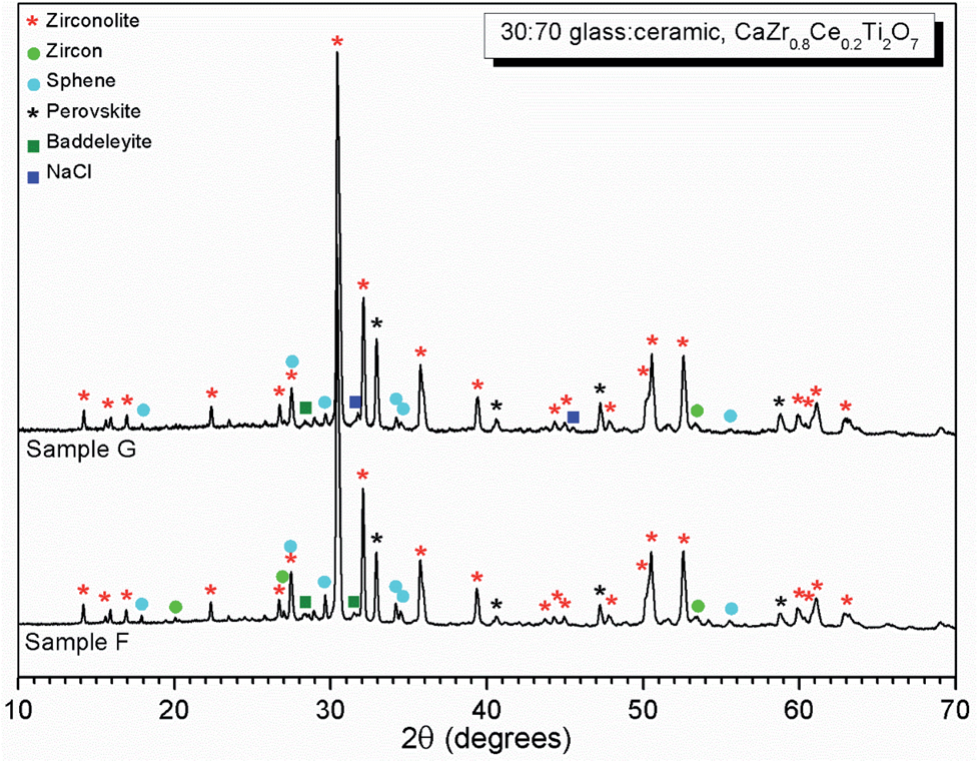
PXRD data of glass–ceramic sample compositions F and G, showing zirconolite (CaZrTi_2_O_7_) as the major crystalline phase with trace zircon (ZrSiO_4_), sphene (CaTiSiO_5_) and baddeleyite (ZrO_2_). Ingrowth of (200) reflection of NaCl, is apparent at 2*θ* = 31.7° in the data of composition G.

**Fig. 11:**
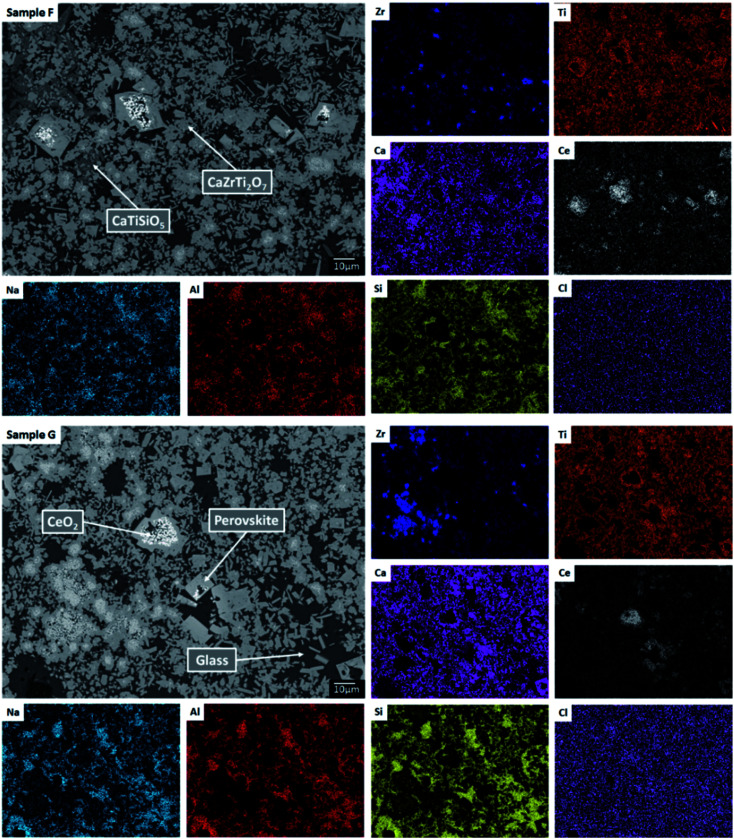
Backscattered electron micrograph and EDX maps for sample compositions F and G demonstrating partitioning of Ce to the zirconolite and perovskite phases, with trace residual CeO_2_.

Two glass–ceramic compositions were fabricated targeting Ce^4+^ incorporation on the Zr^4+^ sites within zirconolite (CaZr_0.8_Ce_0.2_Ti_2_O_7_). These compositions (F and G) were formulated with Cl content of 0.9 wt% and 1.7 wt% Cl, straddling the Cl solubility limit of 1.0 ± 0.1 wt% in the glass phase determined for the baseline compositions (A–E). Fig. 10 and 11 show the PXRD and SEM-EDX data for the two Ce containing samples, respectively. These data show the glass–ceramic phase assemblage and microstructure to be unchanged by addition of CeO_2_ to the formulation. Zirconolite (CaZrTi_2_O_7_) was obtained as the major crystalline phase with minor accessory phases zircon (ZrSiO_4_), sphene (CaTiSiO_5_), baddeleyite (ZrO_2_) and perovskite (CaTiO_3_, PDF card: 01-077-8911) present. The PXRD data of composition G with nominal 1.7 wt% Cl exhibited additional reflections characteristic of NaCl, which were not observed in the PXRD data of composition F with nominal 0.9 wt% Cl. SEM-EDX analysis again demonstrated the partitioning of Cl exclusively to the glass phase with no evidence of incorporation in the zirconolite phase, within detection limits. EDX maps revealed phase separation of NaCl in the microstructure of composition G with nominal 1.7 wt% Cl, but not composition F, with nominal 0.9 wt% Cl, in agreement with PXRD data. The Cl content of the glass phase for both compositions was determined to be 1.0 ± 0.1 wt% (Table 3), consistent with the Cl solubility limit of 1.0 ± 0.1 wt% established for the glass phase in the baseline compositions. Phase separation of NaCl was observed above this threshold for composition G.

#### Cl K-edge X-ray absorption spectroscopy

**Fig. 12:**
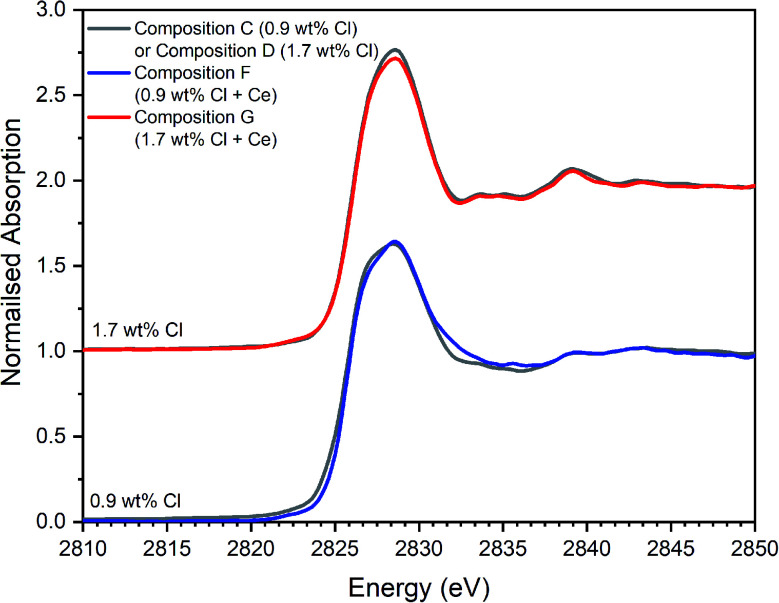
Comparison of merged, background subtracted and normalised Cl K-edge XANES data for the Ce-incorporated glass–ceramic sample compositions F and G, with that of equivalent Ce-free glass–ceramics, C and D from Table 1.

**Fig. 13:**
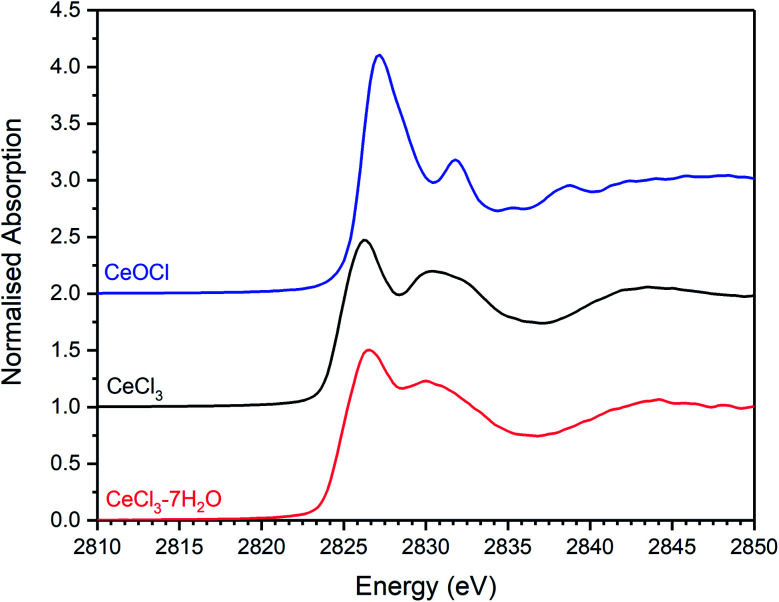
Plot showing the merged, background subtracted and normalised Cl K-edge XANES data for CeCl_3_·7H_2_O, CeCl_3_, and CeOCl reference materials.

Fig. 12 and 13 show the merged, background subtracted and normalised Cl K-edge XANES data for glass–ceramics incorporating Ce as a Pu surrogate, and, separately, data for CeOCl, CeCl_3_ and CeCl_3_·7H_2_O reference compounds; a three point smoothing algorithm was applied to each data set. All data from reference compounds were corrected for self-absorption using the FLUO algorithm.^39^

The Cl XANES data of the Ce-free and Ce-incorporated glass–ceramics were very similar, as demonstrated by the comparison in Fig. 12, for comparable nominal Cl content. The *E*_0_ of Ce-incorporated glass–ceramics was within the range 2825.6–2826.8 eV, previously established for the Ce-free glass–ceramics. The similar XANES features and *E*_0_ of the Ce-free and Ce-incorporated glass–ceramics implies a common primary Cl speciation as the Cl^−^ anion within the aluminosilicate glass phase, in agreement with SEM-EDX analysis (Table 3). Nevertheless, close comparison of the XANES features of Ce-free and Ce-incorporated glass–ceramics showed some subtle differences that suggested potentially different proportions of component Cl environments in the counterpart materials.

Combinatorial linear combination analysis was applied to the Cl K-edge XANES data of the Ce-incorporated glass–ceramic materials, with the aim of identifying and quantifying component model Cl environments, according to the methodology in Section 3.1. This analysis utilised a library of XANES data from 8 reference compounds, comprising: eudialyte, sodalite, chlorellestadite, and afghanite; plus, NaCl, CeOCl, CeCl_3_, and CeCl_3_·7H_2_O.

**Fig. 14:**
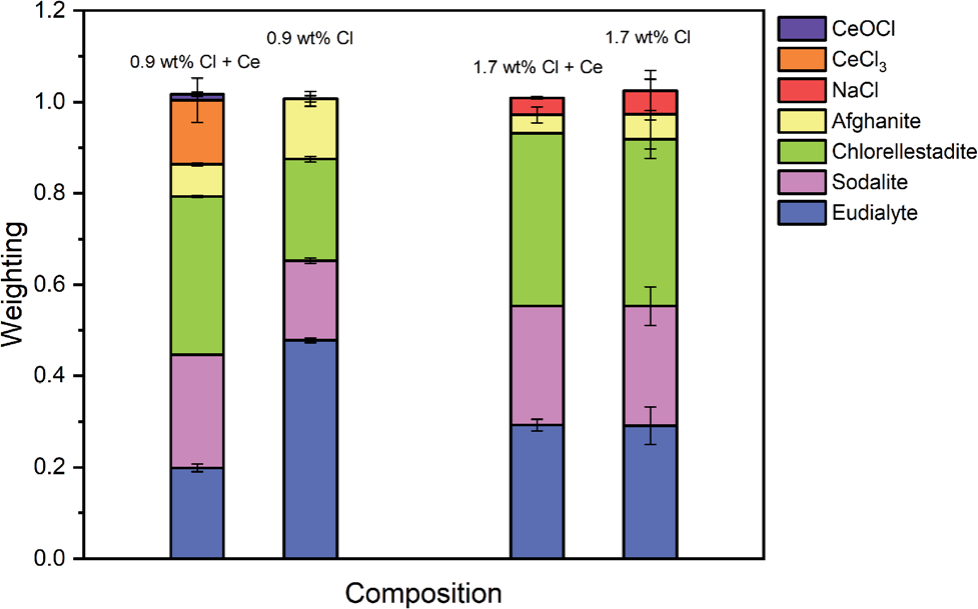
The mean weighted contributions of XANES data from reference compounds NaCl, CeCl_3_, CeOCl, afghanite, chlorellestadite, sodalite and eudialyte, required to fit the glass–ceramics data of glass–ceramic sample compositions F and G; see text for discussion.

Fig. 14 compares the weighted contributions of reference compounds fitted to the Cl XANES data of Ce-free and Ce-incorporated glass–ceramics. The major environments are again of eudialyte, chlorellestadite, and sodalite, with a minor contribution from afghanite and NaCl. The contribution of NaCl was present only in composition G with nominal 1.7 wt% Cl, for which phase separation of crystalline NaCl was detected by PXRD and SEM-EDX. For composition F, with nominal 0.9 wt% Cl, a minor contribution of CeCl_3_ and trace contribution of CeOCl were required to adequately fit the XANES data, implying a small fraction of Cl environments associated with a Ce nearest neighbour.

**Fig. 15:**
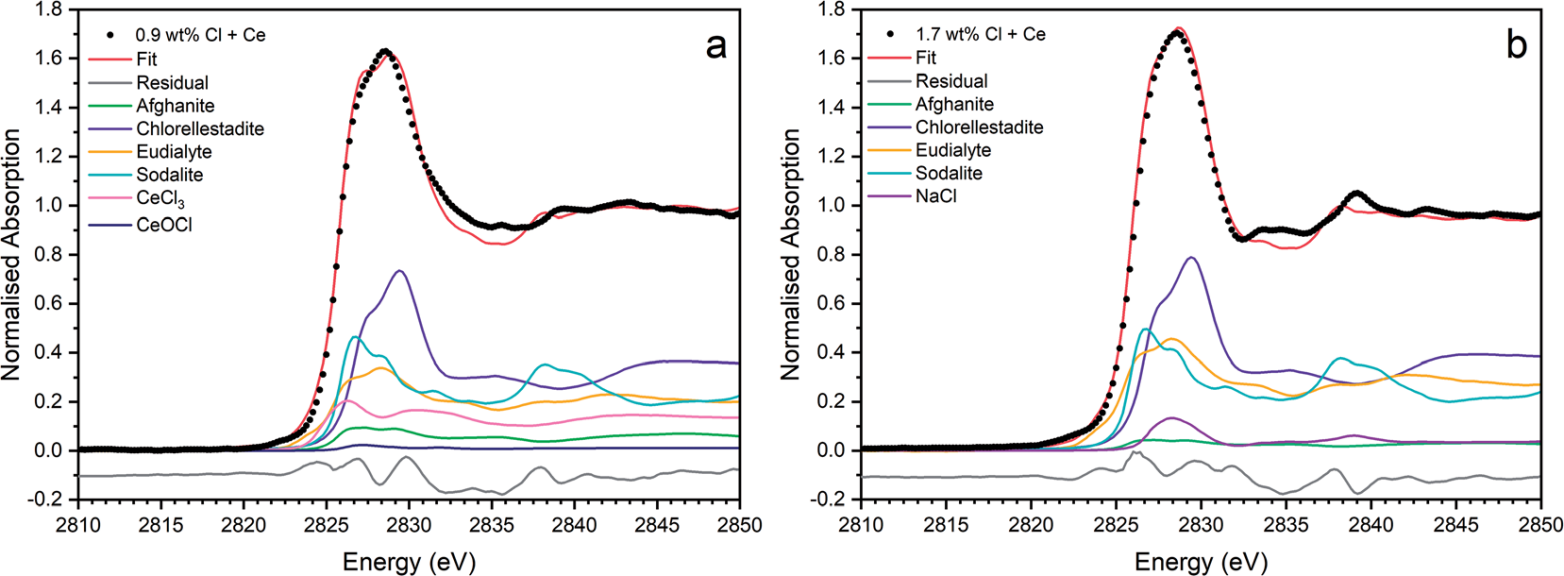
Linear combination fits with the lowest *R* factor for combinatorial fitting of reference spectra to Cl incorporated CeO_2_ doped baseline glass–ceramic sample compositions with (a) 0.9 wt% Cl (composition F) and (b) 1.7 wt% Cl (composition G).

Fig. 15 shows the linear combination fits with the lowest *R* factor for the Ce glass–ceramics with nominal 0.9 wt% and 1.7 wt% Cl. Inspection of the fit and difference profile showed that, although the sum of the 6–7 weighted reference data sets provided a reasonable fit to the observed data, one or more additional components were evidently required for a complete description. Consequently, our interpretation of the model Cl environments present in the glass–ceramics is incomplete, however, the adequacy of the fit enabled consideration of the relative proportion of model Cl environments, though absolute values should be treated with due caution.

## 4. Discussion

Our combined PXRD, SEM-EDX and XANES data demonstrated that Cl partitions exclusively to the aluminosilicate glass phase of the zirconolite glass–ceramic formulations, below a solubility limit of 1.0 ± 0.1 wt% Cl. Above this threshold, phase separation of NaCl occurs, as micron sized crystalline inclusions within the microstructure. The determined Cl solubility limit is within the typical range reported for alkali/alkaline earth aluminosilicate glasses.^16–20^ In this study, NaCl was used as the Cl source, although NaCl is not considered representative of the contaminant Cl species in PuO_2_ residues, which is yet to be identified; however, its use as a Cl source was appropriate for the purpose of establishing the Cl solubility limit in the accessory glass phase and ensured retention during the HIP bake out cycle. The phase assemblage and microstructure of the glass–ceramic formulation proved tolerant to Cl incorporation, with zirconolite formed as the dominant phase and trace zircon, sphene, rutile and baddeleyite present as accessory phases, independent of the Cl content. The addition of Cl to the formulation had no impact on Ce incorporation in the glass–ceramic materials, with Ce partitioning effectively to the zirconolite phase as intended; Ce also partitioned to the perovskite accessory phase present in these formulations. This behaviour was in agreement with Ce partitioning behaviour in glass–ceramic formulations reported previously, without Cl addition.^14,15^

Our preliminary study of PuO_2_ incorporation in the zirconolite glass–ceramic wasteform demonstrated that CeO_2_ is an effective surrogate, achieving comparable partitioning between glass and ceramic phases.^14,15^ The conservative upper bound of Cl contamination in PuO_2_ residue feedstock, would translate into a Cl concentration of *ca.* 1.0 wt% in the aluminosilicate glass phase for a 20 wt% PuO_2_ loading in the ceramic, whilst the typical expected upper limit would yield a Cl concentration of 0.5 wt%, well below the solubility limit established here. The current conceptual process for HIP immobilisation of plutonium residues incorporates provision for a heat treatment facility to remove Cl contaminants prior to immobilisation, due to the uncertainty of Cl behaviour within the wasteform, which would yield a Cl inventory an order of magnitude lower than the quoted upper limits.^26^ However, the results of this study imply that Pu-residues would not necessarily require heat treatment prior to immobilisation to remove the Cl contamination, since the Cl inventory can be accommodated within the aluminosilicate glass phase, even at the upper limit of expectations. Additionally, this research provides evidence to demonstrate compatibility of the wasteform with the expected variation in feed composition, to support the letter of compliance required for immobilisation of Pu-residues in a glass–ceramic wasteform pending disposal in a UK geological disposal facility.^42,43^

Interpretation of Cl K-edge XANES data showed Cl to be speciated as the Cl^−^ anion, primarily co-ordinated to Na and Ca, within the aluminosilicate glass phase, with coexistence of several model environments characteristic of eudialyte, sodalite, chlorellestadite and afghanite (plus CeCl_3_ and CeOCl). Fig. 8 shows an increase in sodalite and chlorellestadite environments, with increasing Cl concentration, at the expense of eudialyte and afghanite environments. Below the Cl solubility limit, the dominant Cl environment is described by eudialyte, in which Cl is co-ordinated (ideally) to 3 × Na cations, encapsulated by a framework comprised of corner sharing SiO_4_, ZrO_6_ and MO_*n*_ polyhedra (*n* = 4, 5, 6, depending on M = Fe, Mn, Nb).^44,45^ The eudialyte structure is known to be highly flexible toward isomorphic substitution, for example Ca for Na, Al for Si, and Ti for Zr.^44,45^ Thus, the local Cl environment in eudialyte is a plausible model for that in the aluminosilicate glass phase. Above the Cl solubility limit, the Cl environment is described by approximately equal proportions of eudialyte, sodalite and chlorellestadite environments. In sodalite, Cl is co-ordinated to 4 × Na cations at the centre of tetrahedral clusters, encapsulated in an aluminosilicate cage;^46^ whereas, in chlorellestadite, Cl is co-ordinated to 3 × Ca cations, within a one dimensional tunnel formed by corner sharing (Si,S,P)O_4_ and CaO_9_ polyhedra, with one short Cl–Cl contact along the tunnel axis.^47^ A minor afghanite contribution was determined for all Cl concentrations; in the afghanite structure, there are three unique Cl environments located within the channels of the aluminosilicate framework, each comprises Cl co-ordinated to 2 × Ca cations.^48^ Our analysis points to the presence of four potential environments within the aluminosilicate glass phase of the glass–ceramics, with Cl co-ordinated to: (a) 3 × Na (similar to eudialyte); (b) 4 × Na (similar to sodalite); (c) 2 × Ca (similar to afghanite); and, (d) 3 × Ca plus 1 Cl (similar to chlorellestadite); however, we cannot rule out the presence of mixed Na/Ca environments, since these are not represented in our reference library.

From Fig. 8, we estimate Cl is co-ordinated, on average, to 3 ± 1 cations, independent of Cl concentration (within precision). Cl has an apparent preference for co-ordination to Ca over Na, given the ratio Na_2_O/CaO = 1.5 on a molar basis in the glass phase, from EDX analysis. Thus, we may conclude that Ca plays an important role in the solubility mechanism of Cl in the glass phase, through formation of Ca–Cl bonds. At low Cl concentrations, below the Cl solubility limit in the glass phase, the major eudialyte environment is correlated with the highest concentration of ZrO_2_ and TiO_2_ in the glass phase. The formation of sodalite and chlorellestadite environments at the expense of eudialyte environments, above the Cl solubility limit, is associated with the lowest concentration of ZrO_2_ and TiO_2_ in the glass phase. Thus, the presence of network forming ZrO_2_ and TiO_2_ in the glass phase also appears to be important in the solubility mechanism of Cl at low concentration, by effectively templating the local glass network to form a eudialyte like environment of corner sharing SiO_4_, ZrO_6_ and TiO_*n*_ polyhedra, encapsulating (ideally) a Na_3_Cl cluster. The increase in sodalite environments close to and above the Cl solubility limit, suggests that formation of Na_4_Cl clusters may be the prelude to phase separation of NaCl.

The Cl environments determined in this study are in reasonable agreement with those identified in previous ^35^Cl MAS-NMR and Cl K-edge XAS studies of aluminosilicate and aluminoborosilicate glasses.^30–32^ Stebbins and Du investigated Cl speciation in sodium aluminosilicate glasses by ^35^Cl MAS-NMR, including the composition NaAlSi_3_O_8_ + 1.5 wt% NaCl (approximately between ideal glass compositions C and D in this study).^31^ The ^35^Cl chemical shift was determined to be intermediate between NaCl and sodalite, which was interpreted as evidence for between 4–6 Na nearest neighbours for Cl. Sandland *et al.* reported a ^35^Cl MAS-NMR study of Na_2_O–CaO–SiO_2_ glasses and concluded from analysis of chemical shift and quadrupolar coupling parameters, the presence of Cl environments with Na, Ca and both Ca and Na neighbours.^32^ Baasner *et al.*, investigated Cl speciation in peralkaline and peraluminous Na_2_O–CaO–Al_2_O_3_–SiO_2_ glasses using ^35^Cl MAS-NMR.^30^ For the peralkaline compositions, most relevant to this investigation, they determined the presence of Cl environments with Na, Ca and mixed Ca and Na neighbours, with a slight preference of Cl for Na over Ca. McKeown *et al.*, applied Cl K-edge XAS to investigate Cl speciation in complex aluminoborosilicate glasses, for radioactive waste immobilisation.^34^ Analysis of both XANES and EXAFS data suggested the dominant Cl environment to be similar to that in the mineral davyne, with Cl co-ordinated to 2 × Ca cations; an additional CaCl_2_ like environment, with Ca co-ordinated to 3 × Ca cations, was inferred, in compositions with high CaO content.^34^ The glass compositions studied by McKeown had Na : Ca ratios in the range 1 < Na_2_O/CaO < 10, which implies an apparent strong preference for Ca over Na.

Our data are consistent with the presence of Cl co-ordination by both Na and Ca in the aluminosilicate glass phase of glass–ceramics, as determined by ^35^Cl MAS-NMR studies of simple Na_2_O–Al_2_O_3_–SiO_2_ and Na_2_O–CaO–Al_2_O_3_–SiO_2_ glasses.^30–32^ We cannot explicitly confirm or exclude the presence of Cl co-ordination by both Ca and Na, which was not an environment characteristic of our library of reference compounds; however, the consensus of ^35^Cl MAS-NMR studies suggests this is likely. In contrast to the conclusion of these ^35^Cl MAS-NMR studies, this investigation points to an apparent preference for Cl co-ordination to Ca over Na in the aluminosilicate glass phases. The aluminosilicate glass phase in this investigation also incorporates significant adventitious ZrO_2_ and TiO_2_, which require charge compensation by Na_2_O. Consequently, the apparent preference of Cl for co-ordination to Ca over Na may arise from the limited availability of Na_2_O as a result of the requirement to charge balance the incorporation of ZrO_2_ and TiO_2_. Such a mechanism could also explain the apparent strong preference of Cl for co-ordination to Ca, rather than Na, observed by McKeown *et al.*,^34^ since the complex waste glass compositions also contained B_2_O_3_ and minor oxides which would similarly require charge compensation by Na_2_O.

The addition of Ce to the glass–ceramic formulation did not change the Cl environments determined to be present in the glass phase, although some minor variation in the relative proportion of environments was observed, consistent with changes in minor oxide concentration, see Table 3. The inference of a minor component of CeCl_3_ environments below the Cl solubility limit is intriguing. Given that association of Ce and Cl was not observed by SEM-EDX, in contrast to phase separated NaCl, this implies Cl association with Ce within the aluminosilicate glass phase. In CeCl_3_, Cl is co-ordinated to 3 × Ce cations at the apex of a flattened tetrahedron,^49^ whereas, in CeOCl, Cl is co-ordinated to 5 × Ce cations at the corners of a square pyramid.^50^ The contribution of the eudialyte environment for the Ce-incorporated glass–ceramics was relatively low and consistent with the lower concentration of ZrO_2_ and TiO_2_ in the glass phase (see Table 3). The significant contribution of the chlorellestadite environment for Ce incorporated glass–ceramics again demonstrated that Ca plays an important role in the solubility mechanism of Cl in the glass phase, through formation of Ca–Cl bonds, consistent with the presence of minor CaO in the glass phase (see Table 3). No significant contribution of CeCl_3_ or CeOCl environments was required to fit the XANES data of the Ce-incorporated glass–ceramic composition with 1.7 wt% Cl, which is consistent with lower Ce content in the glass phase (see Table 3).

Ponader and Brown applied Ln L_3_ edge XAS to investigate the interaction of lanthanides (Ln = La, Gd, Yb) with halogens (Cl or F) in albite, sodium trisilicate, and peralkaline glasses (ideally, NaAlSi_3_O_8_, Na_2_Si_3_O_7_, and Na_3.3_AlSi_7_O_17_, respectively).^51^ In fluoride containing glasses, Ln–F complexes were formed, whereas no evidence was found for Ln–Cl complexes in chloride containing glasses. As demonstrated by Ponader and Brown, the EXAFS is sufficiently sensitive to differentiate LnO_8_ and LnCl_8_ species, due to the large difference in phase shift arising from O and Cl backscattering atoms. Although EXAFS data could be fitted using models involving mixed Ln–O/Cl co-ordination, the goodness of fit was lower than models involving only Ln–O co-ordination. The proportion of Cl–Ce environments determined in our study, *ca.* 15% of total, is consistent with dominant Ln–O co-ordination in chloride bearing aluminosilicate glasses, in agreement with Ponader and Brown and is expected to be at the margin of significance in analysis of Ln L_3_ EXAFS data acquired from such disordered materials. Thus, whilst it is clear that Cl is associated primarily with alkali and alkaline earth network modifiers, within the channels system of aluminosilicate glasses, a minor fraction of Cl associated with lanthanide cations cannot be excluded and would be worthy of further investigation, as would potential minor association between Cl and actinide cations.

## 5. Conclusions

A zirconolite glass–ceramic wasteform suitable for immobilisation of UK plutonium has been demonstrated to be tolerant to incorporation of chlorine impurities present in residue feeds. Chlorine partitions exclusively to the aluminosilicate glass phase, as the chloride anion, below the solubility limit of 1.0 ± 0.1 wt%. This solubility limit is broadly consistent with that reported for natural aluminosilicate glasses. Above this threshold, crystalline NaCl is exsolved as micron sized inclusions within the glass matrix. The incorporation of chlorine does not adversely impact the phase assemblage, microstructure or partitioning of CeO_2_ (as a PuO_2_ surrogate). The established solubility limit exceeds the maximum envisaged chloride inventory in the glass phase (0.5 wt% at a 20 wt% PuO_2_ loading) and is equivalent to the conservative upper bound (1.0 wt% at a 20 wt% PuO_2_ loading), such that blending of heavily and lightly contaminated plutonium residues will not be required.

Cl K-edge XANES demonstrated the mechanism of chlorine incorporation to involve co-ordination to Na and Ca modifier cations, likely in mixed clusters. Combinatorial fitting of a library of chemically plausible reference data, point to the presence of chloride local environments, within the glass phase, characteristic of eudialyte, sodalite, ellestadite and afghanite. The relative proportion of these environments is correlated with the adventitious incorporation of minor Ca, Zr and Ti within the glass phase, which effectively template a compatible local environment.

The current conceptual flowsheet for immobilisation of plutonium residues, by hot isostatic pressing technology, incorporates provision for an upstream heat treatment facility, one purpose of which is to remove chlorine contamination prior to treatment. This study provides confidence that the maximum envisaged chlorine inventory would be significantly below the chlorine solubility limit in the glass phase, without the need for upstream heat treatment or blending of waste feed. Consequently, the heat treatment plant would not be required for the purpose of decontaminating the product feed, although it is likely to be required to underpin long term storage of plutonium residues. Nevertheless, the conceptual immobilisation flowsheet would be considerably de-risked by eliminating the requirement for this facility. Additionally, simplification of the immobilisation flowsheet would be expected to translate into lower capital and recurrent costs.

## Conflicts of interest

There are no conflicts to declare.

## Supplementary Material

RA-010-D0RA04938G-s001
