# Supplementary material for: Solubility, speciation and local environment of chlorine in zirconolite glass–ceramics for the immobilisation of plutonium residues
Source: RSC Adv. 2020 Sep 2;10(54):32497–510. doi: 10.1039/d0ra04938g (PMC9056643; doi:10.1039/d0ra04938g)
Supplement: RA-010-D0RA04938G-s001 [file RA-010-D0RA04938G-s001.pdf]

Electronic Supplementary Material (ESI) for RSC Advances.
This journal is © The Royal Society of Chemistry 2020

Supplementary Data for:

Zirconolite glass-ceramics for the immobilisation of chlorine contaminated plutonium residues.

Stephanie M. Thornber[a], Lucy M. Mottram[a], Amber R. Mason[a], Paul Thompson[b,c], Martin C. Stennett[a], and Neil C. Hyatt[a,*]

[a]*Immobilisation Science Laboratory, Department of Materials Science & Engineering, The University of Sheffield, Sir Robert Hadfield Building, Mappin Street, Sheffield S1 3JD, UK*

[b]*XMaS, UK CRG, ESRF, 71 Avenue des Martyrs, 38043 Grenoble, France.*

[c]*Department of Physics, University of Liverpool, Oliver Lodge Laboratory, Liverpool L69 7ZE, UK.*

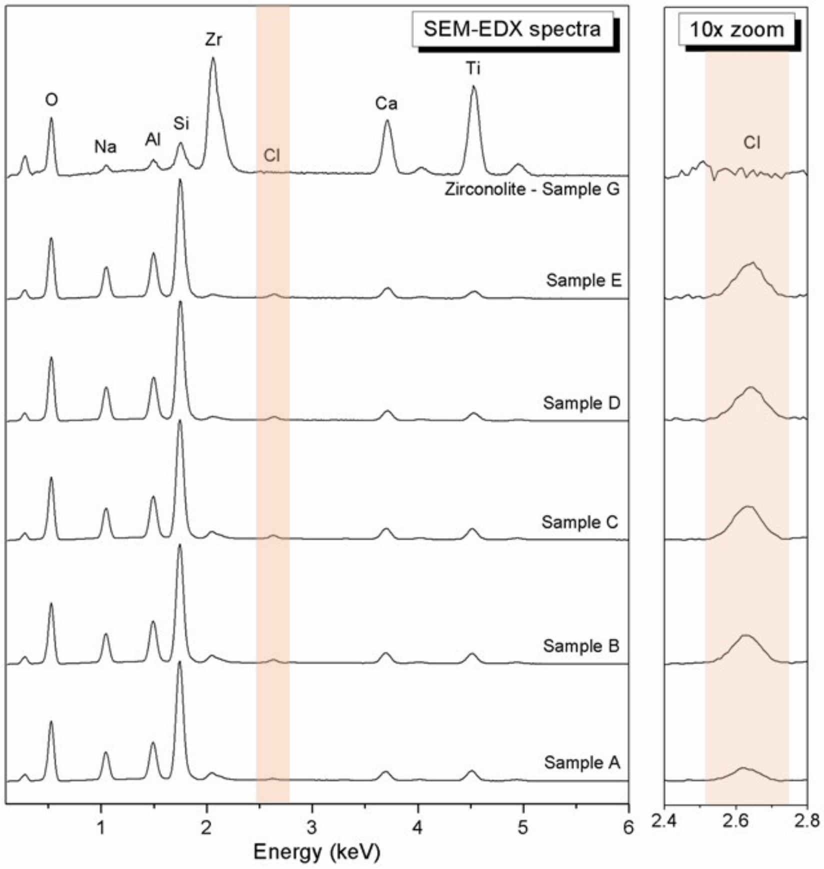

Figure S1: EDX spectra of the glass phase confirming retention and incorporation of Cl and absence of Cl in zirconolite phase.

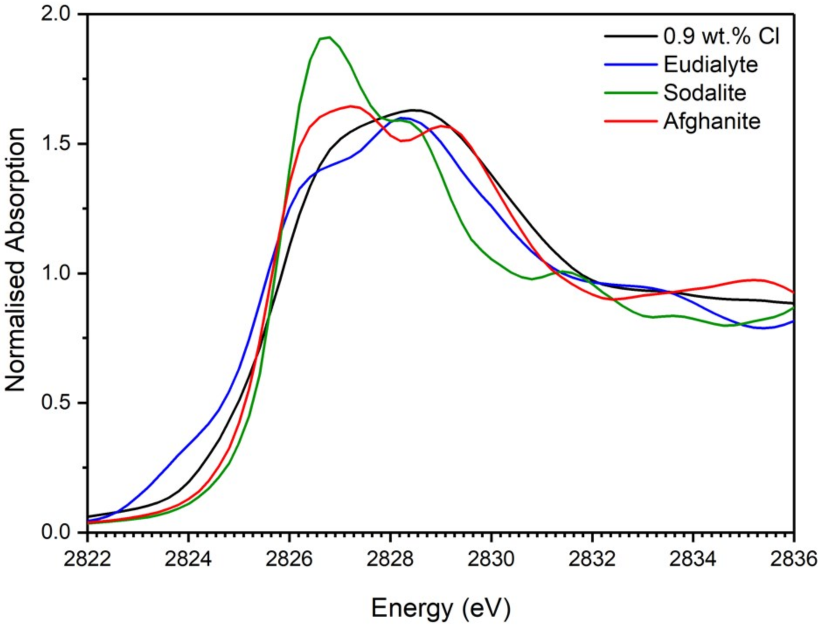

Figure S2: Comparison of Cl K-edge XANES data for glass-ceramic composition C (0.9 wt. % Cl) compared with the XANES data for aluminosilicate reference compounds eudialyte, sodalite and afghanite.
